# Benzimidazole scaffolds as promising antiproliferative agents: a review

**DOI:** 10.1186/s13065-019-0579-6

**Published:** 2019-05-15

**Authors:** Sumit Tahlan, Sanjiv Kumar, Saloni Kakkar, Balasubramanian Narasimhan

**Affiliations:** Faculty of Pharmaceutical Sciences, Maharshi Dayanand University, Rohtak, 124001 India

**Keywords:** Benzimidazole derivatives, Anticancer activity, MTT assay, SRB assay

## Abstract

Cancer is one of the most serious medical problem and second leading cause of death in the world, characterized by a deregulation of the cell cycle which mainly results in a progressive loss of cellular differentiation and uncontrolled cellular growth. The benzimidazole is a heterocyclic moiety found in extensive number of natural and biological active molecules. Benzimidazole derivatives might be considered as auxiliary isosters of nucleotides having attached heterocyclic cores in their structures, cooperate effortlessly with biopolymers and have potential action for chemotherapeutic applications. Benzimidazole and its derivatives displayed a wide range of biological activity because of its structural similarity with the naturally occurring nucleotides. Benzimidazole has established huge alertness in current time and is extremely significant heterocyclic pharmacophore in recent drug innovation and medicinal chemistry. The present review summarizes the chemistry of various substituted benzimidazole derivatives with their antiproliferative significance towards the various cancer cell lines such as HCT116, MCF7, HeLa, HepG2, A549 and A431.
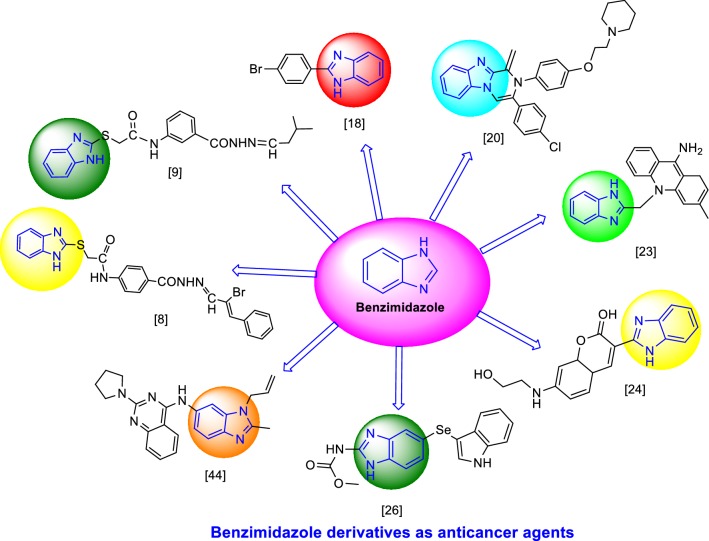

## Introduction

Cancer is one of the most serious medical problem and second leading cause of death in the world, characterized by a deregulation of the cell cycle which mainly results in a progressive loss of cellular differentiation and uncontrolled cellular growth. Hence there is a need to develop those agents whose chemical characteristics clearly differ from those existing agents and can overcome the problem of resistance. In present situation, the most engaged and demanding undertaking is the design, synthesis and development of new biologically active heterocycle compounds. Heterocyclic entities act as medications since they have precise synthetic reactivity and they give advantageous site to which bioactive substituents can be bind. Subsequently, there is need for the improvement of pharmacologically active heterocycles in synthetic and therapeutic science with certain focal points including its effortlessness of activity, greener methodology, simple workup strategy, selectivity, higher yields and high-particle monetary [1, 2].

In the medicinal field, the utility of heterocyclic entities has been raising each day because of structural similarities with biological molecules like nutrients, antibiotics. In spite of the fact that it including almost one-fourth of best hundred offering drugs yet because of issues like obstruction, poisonous quality, there is a requirement for minor change in existing drug molecules and to structure novel molecules which fuse benzimidazole as pharmacophore which are active against new targets [3]. Substituted benzimidazole might be a vital pharmacophore in bioactive agent innovation. Recently, noticeably consideration has been given to the design and synthesis of substituted benzimidazoles. Current perceptions advocate that substituted benzimidazoles and heterocycles demonstrate interface with the biopolymers, have potential action with lower toxicities. The substituted benzimidazoles are helpful for the improvement of ongoing scaffolds of pharmaceutical or natural concern [4].

**Fig. 1:**
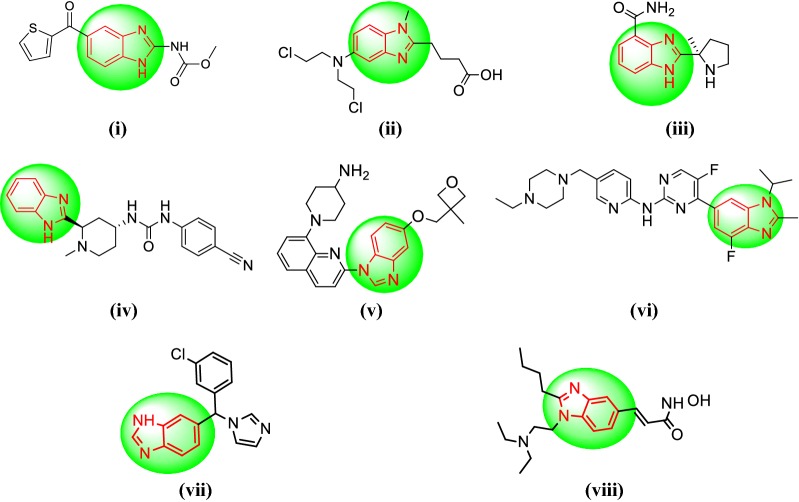
Marketed drugs having benzimidazole moiety

Benzimidazole is also named as 3-azaindole, azindole, benziminazole, benzoglyoxaline, 3-benzodiazole, 1,3-diazaindene having melting point of 170–172 °C and occurs as white crystals [5]. Benzimidazole is an important structural motif found in extensive number of natural and pharmacologically active molecules. Especially, the benzimidazoles might be considered as auxiliary isosters of nucleotides having attached heterocyclic cores in their structures, cooperate effortlessly with biopolymers and have potential action for chemotherapeutic applications [6]. The benzimidazole moiety itself is an urgent pharmacophore in present day and has been used as privileged scaffolds to synthesize selective drugs of interest in numerous therapeutic areas including HIV-RT inhibitor [7], anticancer [8], antimicrobial [9], antihistamine [10], antihelmintic [11], antioxidant [12], antihypertensive [13], antiviral [14], anticoagulant [15] and antiulcer activity [16]. The marketed drugs having benzimidazole moiety (Fig. 1) i.e. (i) nocodazole, (ii) bendamustine, (iii) veliparib, (iv) glasdegib, (v) crenolanib, (vi) abemaciclib, (vii) liarozole, (viii) pracinostat. Malignancy is a gathering of various dangerous ailments described by uncontrolled development of cells, prompting attack of encompassing tissue and regularly spreading to different parts of the body [17]. Development of resistance and toxicity to normal rapidly growing cells are the major limitations of existing anticancer drugs, also majority of the drugs in the market that are not specific [8].

### Benzimidazole derivatives as antiproliferative agents

**Fig. 2:**
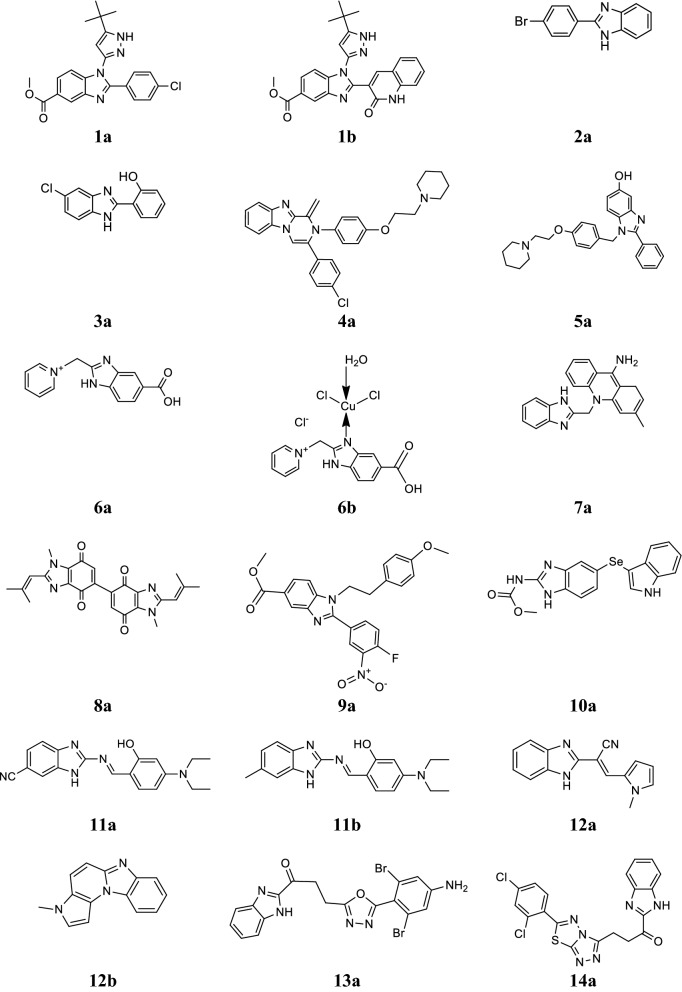
Molecular structures of compounds (**1a**–**1b**, **2a**, **3a**, **4a**, **5a**, **6a**–**6b**, **7a, 8a**, **9a**, **10a**, **11a**–**11b**, **12a**–**12b**, **13a** and **14a**)

Abonia et al. synthesized new derivatives of 1,2,5-trisubstituted benzimidazole and screened for their antiproliferative activity against the 60 human cancer cell lines (leukemia, melanoma, lung, colon, brain, ovary, breast and kidney carcinoma etc.) using SRB protein assay to estimate cell growth. Among the synthesized compounds, compounds **1a** and **1b** (Fig. 2) displayed the utmost potency towards lung, melanoma and leukemia cancer cell lines (GI_50_ values 1.15–7.33 µM and 0.167–7.59 µM), respectively and LC_50_ values more than 100 µM [6].

Azam et al. developed a new series of 2-substituted benzimidazoles and screened for its cytotoxicity against selected human tumor cell lines: leukemia (THP-1), MCF-7, PC-3 and adenocarcinomic alveolar basal epithelial cell line (A-549) by trypan blue exclusion method. Among the synthesized compound, **2a** exhibited promising activity against the tested cancer cell lines (Tables 1 and 2, Fig. 2) [18].

**Table 1:** Percentage growth inhibition of compound **2a**

Compound	Conc. (µM)	Cancer cell lines			
		% Growth inhibition			
		MCF-7	THP-1	PC-3	A-549
**2a**	10	36	39	42	30
	50	93	68	60	70
	100	96	71	81	89
Adriamycin	1	72	–	–	–
Paclitaxel	1	–	–	–	65
Mitomycin	1	–	–	61	–
5-FU	20	–	67	–	–

**Table 2:** Anticancer screening results of compound **2a**

Compound	Cancer cell lines (IC_50_ = µM)			
	MCF-7	THP-1	PC-3	A-549
**2a**	35 ± 2	48 ± 2	46 ± 1	43 ± 2

Coban et al. synthesized a new series of 1*H*-benzimidazole compounds and screened for its cytostatic studies using HeLa, MCF7 and A431 cancer cell lines by MTT assay. Compound **3a** exhibited the most profound cytotoxicity and comparable to standard drug (Table 3, Fig. 2) [19].

**Table 3:** Anticancer screening results of compound **3a**

Compound	Cancer cell lines (IC_50_ = µM)		
	A431	HeLa	MCF7
**3a**	6.16	6.04	6.94
Doxorubicin	0.19	0.16	0.31

Demirayak et al. reported a series of pyrazino[1,2-*a*]benzimidazole derivatives and evaluated for its in vitro anticancer activity against 60 human malignant cell lines: leukaemia (L), melanoma (M), NSCLC, CC, CNSC, OC, RC, PC and BC by SRB protein assay. Among the synthesized compounds, compound **4a** was found to be most active anticancer agent and comparable to standard drugs (Table 4, Fig. 2) [20].

**Table 4:** Antiproliferative activity of compound **4a**

Compound	Cancer cell lines (Log GI_50_)									
	L	NSCLC	CC	CNSC	M	OC	RC	PC	BC	MG-MID
**X**	− 5.48	− 5.17	− 5.11	− 5.12	− 5.08	− 5.18	− 4.99	− 4.49	− 4.79	− 5.09
**Y**	− 6.39	− 6.20	− 6.14	− 6.18	− 6.08	− 6.45	− 6.17	− 6.41	− 6.05	− 6.20
**4a**	− 6.40	− 4.40	− 4.00	− 4.92	− 4.47	− 4.00	− 4.00	− 4.00	− 4.62	− 4.63

X: Melphalan; Y: *cis*-diaminedichloroplatinum

Dettmann et al. developed a new series of 2-phenyl-1-[4-(2-piperidin-1-yl-ethoxy) benzyl]-1*H*-benzimidazole derivatives and evaluated for its cytotoxicity against human MCF-7 and MDA-MB-231 breast cancer cell lines. Among the synthesized derivatives, compound **5a** displayed highest cytostatic effects (T/Ccorr ≈ 0%) and comparable to reference (T/Ccorr = 0–20%) effects at a concentration of 5 µM than the standard drug cisplatin (Fig. 2) [21].

Galal et al. synthesized a new class of benzimidazole-5-carboxylic acid derivatives and evaluated for its anticancer activity (growth inhibitory) against 21 human tumor cell lines (seven colon, eight lung and six gastric) by SRB assay. Compounds **6a** and **6b** showed 10 times superior inhibitory result than etoposide as reference (Table 5, Fig. 2) [22].

**Table 5:** Anticancer activity (growth inhibitory) results of compounds (**6a** and **6b**)

Compounds	GI_50_ (50% cell growth inhibition in µM)
**6a**	0.095
**6b**	0.091
Etoposide	1.3
Doxorubicin	0.065
SN-38	0.066
Cisplatin	3.9

Gao et al. synthesized a novel series of benzimidazole acridine derivatives and evaluated for its in vitro cytotoxicity toward human erythroleukaemia K562 and malignant hepatoma HepG-2 cells by MTT assay. From this series, compound **7a** exhibited maximum cytotoxicity against both K562 (IC_50_ = 2.68 µM) and HepG-2 (IC_50_ = 8.11 µM) cells as compared to standard drugs colchicin (IC_50_ = 1.80 µM for HepG-2) and imatinib (IC_50_ = 0.47 µM for K562) (Table 6, Fig. 2) [23].

**Table 6:** Anticancer activity results of compound **7a**

Compound	Cancer cell lines	IC_50_ (µM)
	U251	2.39
**7a**	A375	3.20
	A172	2.86
	Hela	2.76
	CNE-2	2.62
	U118-MG	1.98

Gellis et al. synthesized novel benzimidazole-4,7-dione molecules and evaluated for their cytotoxicity on colorectal, breast and lung cancer cell lines using MTT assay. Among the synthesized compounds, compound **8a** showed tremendous activity (IC_50_ ± 3 µM) and comparable to mitomycin C with IC_50_ ± 0.9 µM (Fig. 2) [24].

Gowda et al. reported a new series of benzimidazole-5-carboxylic acid derivatives and evaluated for its anticancer activity on K562 and CEM cancer cell using DMSO as vehicle control by the trypan blue and MTT assays. In this series, compound **9a** exhibited maximum apoptosis in leukemic cell accompanying an IC_50_ = 3 µM (Fig. 2) [25].

Guan et al. developed a new class of benzimidazole carbamates with indole moiety and accessed for its antiproliferative activity against three tumor cell lines (SGC-7901, A-549 and HT-1080) using MTT assay. In this series, compound **10a** displayed the highest antiproliferative activity towards selected cancer cell lines (Table 7, Fig. 2) [26].

**Table 7:** Anticancer screening results of compound **10a**

Compound	Cancer cell lines (IC_50_ = µM)		
	SGC-7901	A-549	HT-1080
**10a**	0.098 ± 0.002	0.15 ± 0.05	0.13 ± 0.07
Nocodazole	0.080 ± 0.01	0.12 ± 0.03	0.14 ± 0.005

Hranjec et al. synthesized new benzimidazole substituted Schiff bases and evaluated for their in vitro antiproliferative activity toward human cancer cell lines i.e. HeLa (cervical carcinoma), SW620 (colorectal adenocarcinoma, metastatic), MiaPaCa-2 (pancreatic carcinoma), MCF-7 (breast epithelial adenocarcinoma, metastatic) and WI38 (normal diploid human fibroblasts) by MTT assay. From the synthesized compounds, compounds **11a** and **11b** displayed highest antiproliferative activity (Table 8, Fig. 2) [27].

**Table 8:** Anticancer screening results of compounds (**11a** and **11b**)

Compounds	Cancer cell lines (IC_50_ = µM)				
	HeLa	MCF-7	SW620	MiaPaCa-2	W138
**11a**	4.73	9.23	49.15	27.92	0.96
**11b**	3.24	15.27	52.04	22.24	1.67

Hranjec et al. synthesized a new series of novel benzimidazole derivatives and evaluated for its antiproliferative activity on five different cancer cell lines: HeLa, pancreatic (MiaPaCa-2), colon (SW 620), MCF-7 and lung (H 460) cell lines by MTT assay. Among them, compounds **12a** and **12b** displayed the highest activity on tested cell lines and demonstrated an exceptional selectivity for HeLa cells (Table 9, Fig. 2) [28].

**Table 9:** Anticancer activity results of compounds (**12a** and **12b**)

Compounds	Cancer cell lines (IC_50_ = µM)				
	HeLa	MiaPaCa-2	SW 620	MCF-7	H 460
**12a**	0.8 ± 0.4	4 ± 2	30 ± 5	13 ± 3	26 ± 13
**12b**	0.7 ± 0.2	4 ± 2	25 ± 4	11 ± 1	22 ± 2
Cisplatin	3 ± 0.6	4 ± 3	4 ± 1	12 ± 6	0.3 ± 0.04
Doxorubicin	0.04 ± 0.009	0.01 ± 0.01	0.02 ± 0.01	0.04 ± 0.01	Not tested

Husain et al. synthesized a new class of benzimidazole having oxadiazole and triazolo-thiadiazoles moiety and evaluated for its in vitro anticancer potential at concentration (10 µM) against NCI 60 cell lines by five dose assay. Compound **13a** displayed considerable growth reticence with GI_50_ efficacy from 0.49 to 48.0 µM especially in lung carcinoma cell HOP-92 (GI_50_ 0.49, TGI 19.9, LC_50_ > 100 and Log_10_GI_50_ − 6.30, Log_10_TGI − 4.70, Log_10_LC_50_ > − 4.00) (Fig. 2) [29].

Husain et al. synthesized benzimidazole derivatives associated with triazolo-thiadiazole and triazolo-thiadiazine nucleus and screened for their in vitro anticancer potential at only concentration (10^−5^ M) toward NCI 60 cell lines by five dose assay. Among the synthesized compounds, compound **14a** (Fig. 2) exhibited excellent results against 60 cell panel (MG-MID − 6.07, − 5.51 and − 4.85 value of log_10_ GI_50_, log_10_ TGI and log_10_ LC_50_, respectively) [30].

**Fig. 3:**
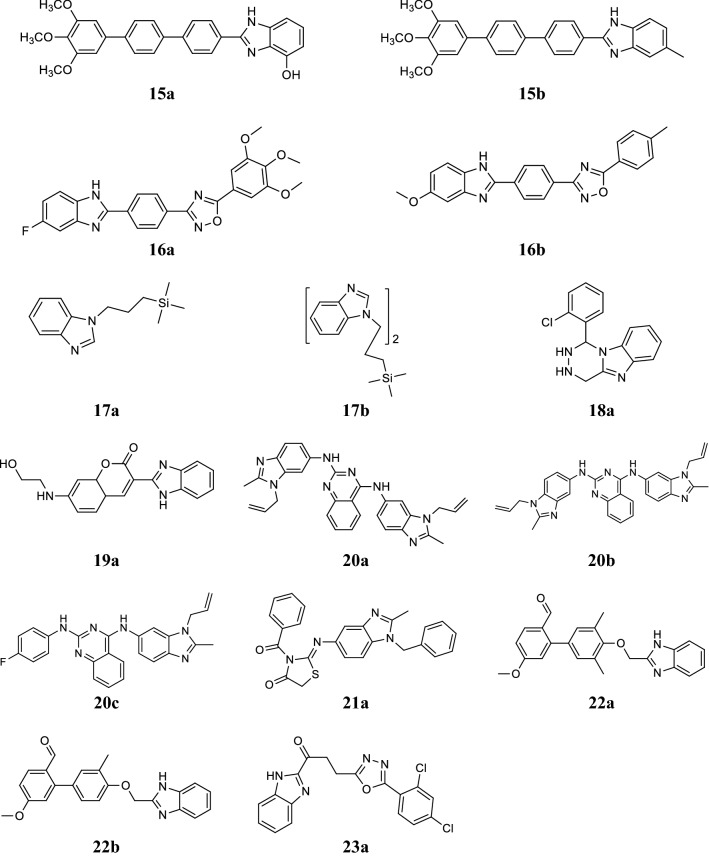
Molecular structures of compounds (**15a**–**15b**, **16a**–**16b**, **17a**–**17b**, **18a**, **19a**, **20a**–**20c**, **21a**, **22a**–**22b** and **23a**)

Kamal et al. synthesized novel terphenyl benzimidazole derivatives and screened for their antitumor potency in tumor cells i.e. oral, lung, ovarian, cervix, colon, breast and prostate cells by SRB method. Among the synthesized compounds, compounds **15a** and **15b** showed significant anticancer potency with GI_50_ values vary from < 0.1 to 2.11 µM, whereas the positive control reference adriamycin demonstrated the GI_50_ value from 0.1 to 7.25 µM (Fig. 3) [31].

Kamal et al. synthesized novel 2-aryl 1,2,4-oxadiazolo-benzimidazole compounds and evaluated for their in vitro anticancer screening against 60 tumor cell lines by SRB method. In this series, compounds **16a** and **16b** displayed significant cytoxicity against the majority of tumor cells with GI_50_ range from 0.79 to 28.2 µM (Fig. 3) [32].

Lukevics et al. developed novel trimethylsilylpropyl substituted benzimidazole derivatives and screened for their anticancer activity on mouse hepatoma (MG-22A), human fibrosarcoma (HT-1080), mouse melanoma (B16), mouse neuroblastoma (Neuro 2A) and normal mouse fibroblast cells by MTT assay. In this series, compounds, **17a** and **17b** showed significant activity in mouse melanoma (B16) having TD_50_ from 0.001 to 0.008 µg/mL. In vivo screening of compound **17a** showed high anticancer activity toward sarcoma S-180 by 62% (Fig. 3) [33].

El-Nassan, synthesized a new series of benzimidazole derivatives and demonstrated for its in vitro anticancer activity on MCF7 by SRB assay. Among the synthesized derivatives, compound **18a** (IC_50_ = 0.0390 µM) exhibited promising antitumor activity (Fig. 3) [34].

Paul et al. synthesized novel coumarin–benzimidazole conjugates and tested for their in vitro anticancer potency on 60 cancer cell lines by SRB assay. In this series, compound **19a** was found to be most active agent against leukemia, breast, colon, prostate (PC-3) and melanoma (LOX IMVI) cancer cell lines, respectively and comparable to the standard drug (5-FU) (Table 10, Fig. 3) [35].

**Table 10:** Percentage growth inhibitory value of compound 19a

Cancer cell lines		Compound 19a	5-Fluorouracil
Leukemia			
HL-60 (TB)		80.51	47.9
CCRF-CEM		72.52	57.1
K-562		57.34	42.3
MOLT-4		38.03	43.1
RPMI-8226		46.65	41.4
Breast tumor			
T-47D		70.68	56.7
MDA-MB231/ATCC		58.91	78.1
MDA-MB-468		48.37	Not tested
BT-549		33.10	37.8
Colon tumor			
HCT-116		62.25	17.8
HCT-15		72.67	26.5
Melanoma cancer LOX IMVI		54.29	30.4
Prostate cancer PC-3		56.69	58.2

Paul et al. designed and synthesized novel quinazoline and benzimidazole conjugates and evaluated in vitro for their antitumor activity on 60 human tumor cell lines at a dose of 10 µM. From this series, compounds **20a**, **20b** and **20c** were found to be most active against selected cancer cell lines (Table 11, Fig. 3) [36].

**Table 11:** Antitumor activity results of compounds (**20a**–**20c**)

Compounds	Activity (µM)	MG-MID
**20a**	GI_50_	1.64
	TGI	3.28
	LC_50_	5.50
**20b**	GI_50_	0.81
	TGI	2.08
	LC_50_	4.47
**20c**	GI_50_	4.52
	TGI	15.9
	LC_50_	57.1
Quinazoline analogue	GI_50_	16.9
	TGI	40.5
	LC_50_	> 100
Benzimidazole analogue	GI_50_	18.1
	TGI	33.4
	LC_50_	56.7

Ramla et al. synthesized a novel series of benzimidazole derivatives and evaluated for its inhibitory activity against Burkitt’s lymphoma by Epstein–Barr virus activation test. In this series, compound **21a** exhibited 12.3% inhibitory activity (Fig. 3) [37].

Ranganatha et al. synthesized new benzophenone–benzimidazole derivatives and evaluated for their in vivo tumor inhibition against EAC cells via three independent assays (trypan blue dye exclusion, MTT and LDH release assay) using DMSO as a vehicle control. Compounds, **22a** and **22b** exhibited the highest cytotoxic effect (IC_50_ ~ 10 μM and ~ 16 μM) among the synthesized derivatives (Fig. 3) [38].

Rashid et al. synthesized benzimidazoles with oxadiazole nucleus and evaluated for their in vitro anticancer activity at a single dose (10 µM) in NCI 60 cell line panel using SRB assay. In this series, compound **23a** with GI_50_ values between 0.79 and 17.8 µM showed significant anticancer activity against tested cell lines (Fig. 3) [17].

**Fig. 4:**
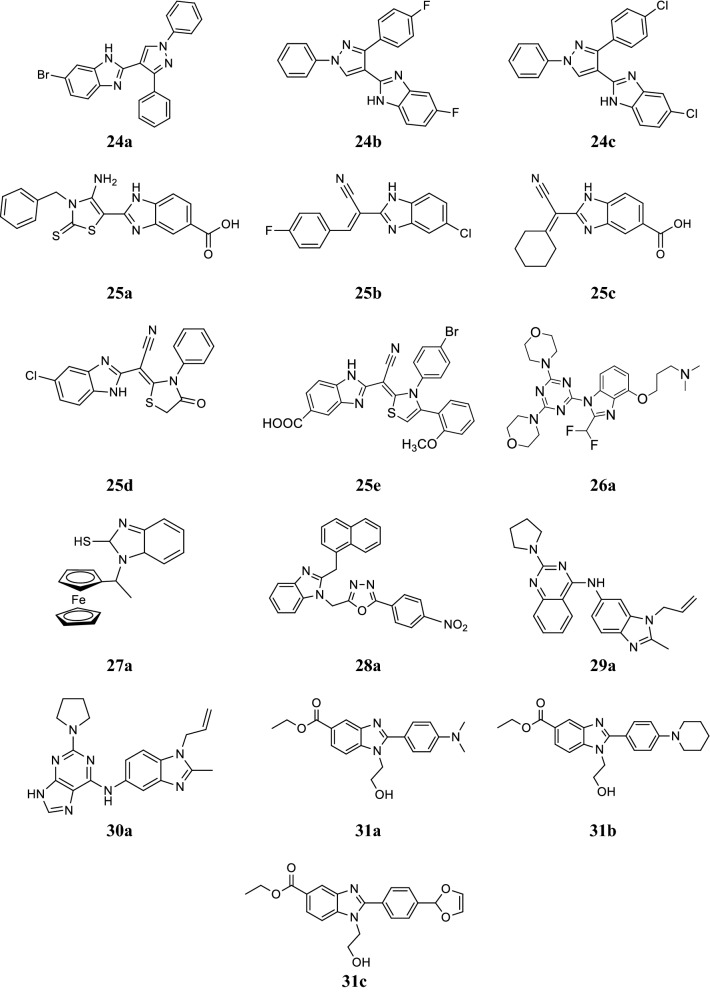
Molecular structures of compounds (**24a**–**24c**, **25a**–**25e**, **26a**, **27a**, **28a, 29a**, **30a** and **31a**–**31c**)

Reddy et al. synthesized novel pyrazole containing benzimidazole conjugates and screened for their anticancer activity (growth inhibition) against lung-A549, MCF-7, HeLa and human keratinocyte cells-HaCaT using MTT assay. Among the synthesized derivatives, compounds **24a**, **24b** and **24c** exhibited effective anti-proliferative activity toward cancer tested cell lines (Table 12, Fig. 4) [39].

**Table 12:** Anticancer activity results of compounds (**24a**–**24c**)

Compounds	Cancer cell lines (IC_50_ µM)			
	A549	MCF-7	HeLa	HaCaT
**24a**	1.81	0.83	1.76	> 50
**24b**	1.13	0.95	1.57	> 50
**24c**	1.34	1.17	1.63	> 50
5-Fluorouracil	2.13	2.36	4.6	15.26
Nocodazole	1.87	1.6	2.83	8.9

Refaat et al. synthesized a novel series of 2-substituted benzimidazole derivatives and evaluated in vitro for its anticancer potency against HEPG2, MCF7 and HCT116 cell lines by SRB assay using doxorubicin as reference. Among the synthesized compounds, compounds **25a** and **25b** showed the highest potency against HEPG2 while compounds, **25c**, **25d** and **25e** showed promising activity against MCF7. Compounds, **25d** and **25e** showed moderate activity against HCT116 (Table 13, Fig. 4) [40].

**Table 13:** Anticancer activity results (IC_50_ and IC_90_ µM) of compounds (**25a**–**25e**)

Compounds	Cancer cell lines	IC_50_	Doxorubicin	IC_90_	Doxorubicin
**25a**	HEPG2	0.55 ± 0.05	0.59 ± 0.03	7.53 ± 0.06	6.82 ± 0.06
**25b**	HEPG2	0.55 ± 0.03	0.59 ± 0.03	7.62 ± 0.09	6.82 ± 0.06
**25c**	MCF7	2.15 ± 0.04	0.72 ± 0.08	11.70 ± 0.17	8.77 ± 0.06
**25d**	MCF7	2.83 ± 0.03	0.72 ± 0.08	12.63 ± 0.09	8.77 ± 0.06
	HCT 116	3.72 ± 0.03	0.65 ± 0.09	12.02 ± 0.07	7.32 ± 0.09
**25e**	MCF7	2.85 ± 0.15	0.65 ± 0.09	13.25 ± 0.13	8.77 ± 0.06
	HCT 116	3.75 ± 0.16	0.72 ± 0.08	12.05 ± 0.06	7.32 ± 0.09

Rewcastle et al. synthesized a series of benzimidazole analogs and evaluated for its enzyme activity against the p110α, β and δ isoforms of PI3K using a lipid kinase assay and also assessed for their antitumor activity against two human cancer cells lines, NZOV9 (Y1021C mutation of p110α enzyme) and NZB5 (wild-type p110α enzyme) using cell proliferation assay. From this series, compound **26a** exhibited best enzyme potency and also inhibiting tumor growth by 56.3 ± 2.6% (Table 14, Fig. 4) [41].

**Table 14:** Anticancer activity results (enzyme and cellular inhibition) of compound **26a**

Compound	p110α IC_50_ (nm)	p110β IC_50_ (nm)	p110δ IC_50_ (nm)	NZB5 IC_50_ (µm)	NZOV9 IC_50_ (µm)
**26a**	11	7.3	4.5	0.17	0.04

Rodionov et al. synthesized novel ferrocenylalkyl 2-mercaptobenzimidazole derivatives and screened for their in vivo antitumor activity against the murine solid tumor, carcinoma 755 (Ca755), transplanted in mice. Among the synthesized compounds, compound **27a** showed 87% tumor growth inhibition on carcinoma 755 at the dose of 250.0 mg/kg day as compared to control cisplatin (Fig. 4) [42].

Salahuddin et al. synthesized a novel series of benzimidazole molecules and screened for its in vitro anticancer activity on leukemia, melanoma, lung, colon, CNS, ovarian, renal, prostate and breast cancer cell lines. From this series, compound **28a** displayed promising activity against MDA-MB-468 (breast cancer) and SK-MEL-28 (melanoma) (GP = 36.23 and 47.56, respectively) (Fig. 4) [43].

Sharma et al. synthesized new benzimidazole–quinazoline conjugates and monitor for their growth inhibitory activity on 60 tumor cell lines. Among them, compound **29a** exhibited superior activity on leukemia, colon and melanoma cancer cell lines as compared to standard 5-fluorouracil (Table 15, Fig. 4) [44].

**Table 15:** Percentage growth inhibitory results (GI %) of compound **29a**

Cancer cell lines	Compound 29a	5-Fluorouracil
Leukemia		
K-562	98.0	42.3
MOLT-4	50.0	43.1
RPMI-8226	45.0	41.4
SR	94.2	24.8
Colon		
HCC-2998	76.6	Lethal
HCC-116	80.3	17.8
HT29	94.3	27.1
Melanoma		
LOX IMVI	97.5	30.4

Sharma et al. synthesized novel purine-benzimidazole conjugates then screened for their anticancer activity against 60 human malignant cell lines by Aurora-A kinase assay. Among them, compound **30a** exhibited 1.25 fold more activity with GI_50_ value of 18.12 µM (MG-MID) than the reference 5-FU, GI_50_ = 22.60 µM (Fig. 4) [45].

Yoon et al. synthesized a new class of benzimidazole derivatives and evaluated in vitro for its antiproliferative activity using human breast cancer MCF-7 and MDA-MB-468 cells by inner salt assay. From this series, compounds **31a**, **31b** and **31c** showed good antiproliferative activity against MCF-7 and MDA-MB-468 cells (Table 16, Fig. 4) [46].

**Table 16:** Anticancer activity (% cell inhibition) of compounds (**31a**–**31c**)

Compounds	Cancer cell lines	
	MCF-7	MDA-MB-468
**31a**	49.63	46.33
**31b**	42.37	45.51
**31c**	62.43	42.30
Cambinol	38.26	22.09

**Fig. 5:**
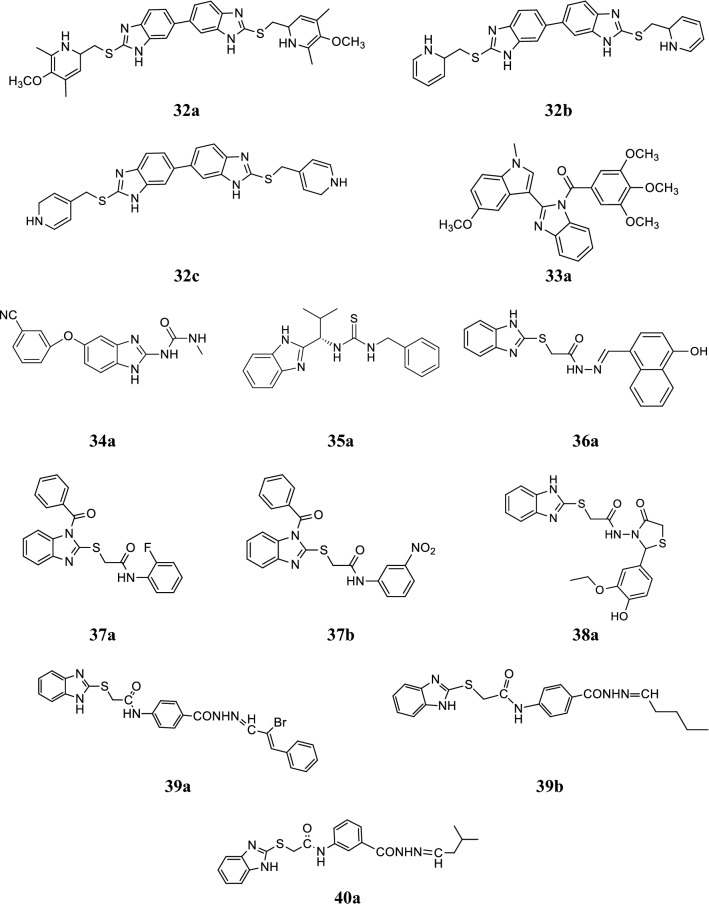
Molecular structures of compounds (**32a**–**32c**, **33a**, **34a**, **35a**, **36a, 37a**–**37b**, **38a**, **39a**–**39b** and **40a**)

Yang et al. synthesized new symmetrical bis-benzimidazoles derivatives and evaluated in vitro for their cytotoxicity on HeLa, SKOV-3 and BGC-823 cell lines by MTT assay. In this series, compounds **32a**, **32b** and **32c** displayed significant activity against tested cancer cell lines (Table 17, Fig. 5) [47].

**Table 17:** Anticancer screening results of compound (**32a**–**32c**)

Compounds	Cancer cell lines (IC_50_ µM)		
	SKOV-3	HeLa	BGC-823
**32a**	2.95	> 50	> 50
**32b**	38.60	7.1	16.4
**32c**	2.81	32.4	11.0
Cisplatin	–	1.6	1.3
Taxol	0.00134	–	–

Wang et al. synthesized new chain of benzene acyl-2-(1-methylindol-3-yl)-benzimidazole derivatives and screened for its tubulin polymerization inhibitory activity and cytotoxicity against anthropic A549, HepG2 and MCF-7 tumor cell lines by MTT assay. Among the synthesized derivatives, compound **33a** displayed excellent activity and comparable to colchicine and CA-4 as standards (Table 18, Fig. 5) [48].

**Table 18:** Cytotoxicity and tubulin polymerization inhibition of compound **33a**

Compound	Cancer cell lines (GI_50_ µM)			Inhibition of tubulin polymerization
	HepG2	A549	MCF-7	(IC_50_ µM)
**33a**	3.8	2.4	5.1	1.5
**CA-4**	7.4	2.8	9.4	1.8
Colchicine	10.5	4.4	13.5	2.62

Wang et al. reported novel benzimidazole-2-urea derivatives and tested for their antiproliferative activity against a group of human tumor cells using MTT assay. In this series, compound **34a** exhibited the potent antiproliferative activity and compared to standard drugs (Table 19, Fig. 5) [49].

**Table 19:** Anticancer activity results (IC_50_ µM) of compound **34a**

Compound	Cancer cell lines						
	NCI-H460	Colo205	K562	A431	HepG2	Hela	MDA-MB-435S
**34a**	0.040	0.050	0.006	0.026	1.774	0.452	0.052
Colchicine	0.021	0.003	0.001	0.008	1.710	0.704	0.007
Taxol	0.010	0.003	0.004	0.007	0.990	0.410	0.009

Madabhushi et al. synthesized some new benzimidazole functionalized chiral thioureas and screened for their cytotoxic activity against the human cancer cell lines (A549, MCF7, DU145 and HeLa) by MTT assay. From the synthesized compounds, compound **35a** found to display significant activity against A549, DU145 and HeLa cell lines (Table 20, Fig. 5) [50].

**Table 20:** Anticancer activity results IC_50_ (µM) of compound **35a**

Compound	Cancer cell lines			
	A549	MCF7	DU145	HeLa
**35a**	5.2	9.8	12.3	11.1
Doxorubicin	0.8	0.7	0.8	0.6

Yadav et al. designed and synthesized a series of new benzimidazole derivatives and accessed for its cytotoxic potential against MCF7 (human breast adenocarcinoma cancer) cell line by SRB technique and compared to 5-FU and carboplatin standard drugs. In this series, compound **36a** displayed the most potent anticancer activity (Table 21, Fig. 5) [51].

**Table 21:** Anticancer activity results of synthesized compound **36a**

Compound	Cancer cell line (IC_50_ = μM)
	MCF7
**36a**	0.0013
**5-FU**	0.0461
Carboplatin	0.2694

Yadav et al. synthesized some 2-(1-benzoyl-1*H*-benzo[*d*]imidazol-2-ylthio)-*N*-substituted acetamide derivatives and evaluated for their anticancer activity against MCF7 and HCT116 cancer cell lines by SRB assay using tamoxifen and 5-FU as references. Among the synthesized compounds, compounds **37a** and **37b** emerged out as excellent anticancer agents (Table 22, Fig. 5) [52].

**Table 22:** Anticancer screening results of compounds (**37a** and **37b**)

Compounds	Cancer cell lines (IC_50_ = μM/mL)	
	MCF7	HCT116
**37a**	0.0047	0.0839
**37b**	0.0786	0.0058
Tamoxifen	0.0043	–
5-FU	–	0.0125

Yadav et al. synthesized a class of novel benzimidazole derivatives and screened for its antitumor potency towards HCT116 cancer cell line by SRB method and comparable to standard drug 5-FU. Compound **38a** showed prominent antitumor activity (Table 23, Fig. 5) [53].

**Table 23:** Anticancer screening results of compound **38a**

Compound	Cancer cell line (IC_50_ = μM/mL)
	HCT116
**38a**	0.00005
5-FU	0.00615

Tahlan et al. synthesized a series of new 2-mercaptobenzimidazole Schiff base derivatives and evaluated for its antitumor potency against HCT116 cancer cell line by SRB method using 5-FU as reference. In this series, compounds **39a** and **39b** showed significant antitumor activity towards tested cell line (Table 24 and Fig. 5) [8].

**Table 24:** Anticancer activity results of synthesized compounds (**39a** and **39b**)

Compounds	Cancer cell line (IC_50_ = μg/mL)
	HCT116
**39a**	8
**39b**	7
5-FU	2.63

Tahlan et al. reported a class of novel benzimidazole azomethine derivatives and screened for its anticancer potency against HCT116 cancer cell line by SRB method using 5-FU as standard. Among the synthesized compounds, compound **40a** was found to be most potent anticancer agent against selected cancer cell line (Table 25 and Fig. 5) [9].

**Table 25:** Anticancer activity results of synthesized compound **40a**

Compound	Cancer cell line (IC_50_ = μg/mL)
	HCT116
**40a**	30
5-FU	0.85

**Fig. 6:**
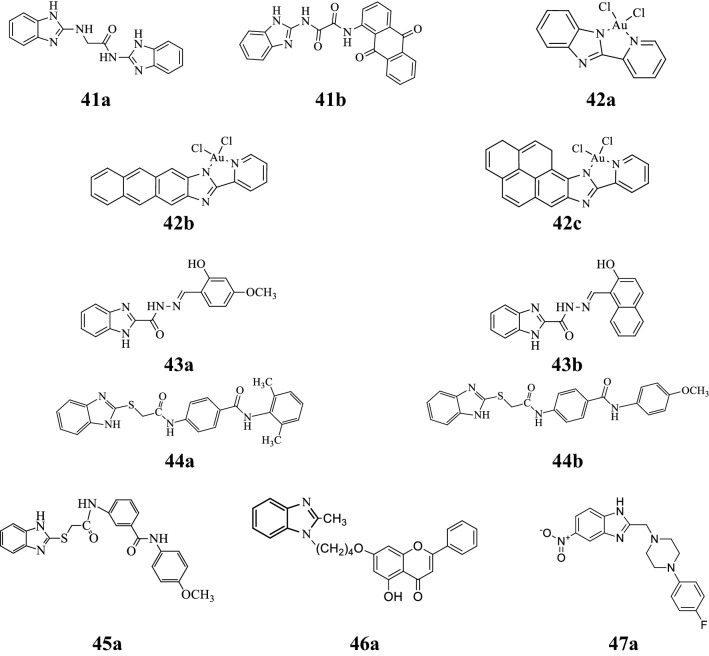
Molecular structures of compounds (**41a**–**41b**, **42a**–**42c**, **43a**–**43b**, **44a**–**44b**, **45a**, **46a** and **47a**)

Mohammed et al. synthesized a class of new substituted benzimidazoles and screened for its anticancer activity against breast adenocarcinoma MCF-7, lung carcinoma A549 and epithelioid cervix carcinoma HeLa using SRB colorimetric assay. Among the synthesized compounds, compounds **41a** and **41b** were found to be most active anticancer agents and comparable to the cisplatin (reference drug) (Table 26, Fig. 6) [54].

**Table 26:** Percentage inhibition results of tested compounds (**41a** and **41b**)

Compounds	Cancer cell lines		
	MCF-7	HELA	A549
**41a**	95	54	77
**41b**	80	35	72
Cisplatin	60	35	60

Aikman et al. developed some gold(III) pyridine-benzimidazole complexes and evaluated for their antitumor activity against human SKOV-3, A375, MCF-7 and A549 cancer cell lines by MTT assay using Auphen (stock solution 10 mM in DMSO) as reference. Compounds **42a**–**42c** showed promising anticancer activity, particularly in the melanoma A375 cancer cell line (Table 27, Fig. 6) [55].

**Table 27:** Anticancer activity results of synthesized compounds (**42a**–**42c**)

Compounds	Cancer cell lines (EC_50_ (µM))			
	SKOV-3	A375	MCF-7	A549
**42a**	17 ± 7	5 ± 2	12 ± 1	> 50
**42b**	33 ± 5	12 ± 2	29 ± 8	> 50
**42c**	41 ± 13	13 ± 2	17 ± 3	45 ± 3
Auphen	7.00 ± 2.00	1.7 ± 0.3	3.00 ± 0.05	1.07 ± 0.09

Onnis et al. synthesized a series of novel benzimidazolehydrazones and evaluated for its anticancer activity against murine leukemia (L1210), T-lymphoblastic leukemia (CEM), cervix carcinoma (HeLa) and pancreas carcinoma (Mia Paca-2) cell lines. In this series, compounds **43a** and **43b** inhibited the growth of all tested cell lines (Table 28, Fig. 6) [56].

**Table 28:** Anticancer screening results of compounds (**43a** and **43b**)

Compounds	Cancer cell lines (IC_50_ = µM)			
	L1210	CEM	HeLa	Mia Paca-2
**43a**	1.6 ± 0.9	0.98 ± 0.02	4.0 ± 0.4	6.3 ± 3.2
**43b**	2.9 ± 1.3	1.0 ± 0.01	2.5 ± 1.4	7.9 ± 0.3

Tahlan et al. designed and synthesized a series of substituted benzimidazole benzamide derivatives and screened for its anticancer potency against HCT116 cancer cell line by SRB method using 5-FU as standard. In this series, compound **44a** and **44b** were found to be most potent compounds against tested cell line (Table 29, Fig. 6) [57].

**Table 29:** Anticancer activity results of synthesized compounds (**44a** and **44b**)

Compounds	Cancer cell line (IC_50_ = μM)
	HCT116
**44a**	5.85
**44b**	4.53
5-FU	9.99

Tahlan et al. designed and synthesized some novel benzimidazole derivatives and accessed for their antiproliferative potential towards HCT116 cancer cell line by SRB method. Among the synthesized derivatives, compound **45a** displayed the most potent anticancer activity (Table 30, Fig. 6) [58].

**Table 30:** Anticancer activity results of synthesized compound (**45a**)

Compound	Cancer cell line (IC_50_ = μM)
	HCT116
**45a**	4.12
5-FU	7.69

Wang et al. developed a class of novel substituted benzimidazole derivatives and evaluated its antiproliferative activity against MGC-803, MCF-7, HepG2 and MFC cells by MTT colorimetric assay. In this class, compound **46a** showed remarkable anticancer activity as compared with standard drugs 5-FU and chrysin (Table 31, Fig. 6) [59].

**Table 31:** Anticancer activity results of synthesized compound (**46a**)

Compound	Cancer cell lines (IC_50_ = μM)			
	MGC-803	MCF-7	HepG2	MFC
**46a**	36.66 ± 4.76	73.21 ± 2.41	53.25 ± 3.26	25.72 ± 3.95
5-FU	74.39 ± 2.03	57.09 ± 3.17	63.37 ± 2.52	78.52 ± 3.92
Chrysin	> 100	> 100	73.29 ± 3.81	95.64 ± 5.04

El-Gohary et al. designed and synthesized a class of novel benzimidzole scaffolds and screened for its in vitro antiproliferative activity against three different cancer cell lines i.e. HepG2, HCT-116, MCF-7 and normal (W138) cell lines employing MTT assay. Among the synthesized compounds, compound **47a** displayed significant antitumor activity and comparable to standard 5-FU (Table 32, Fig. 6) [60].

**Table 32:** In vitro anticancer activity results of synthesized compound (**47a**)

Compound	Cancer cell lines (IC_50_ = mM)			
	HepG2	HCT-116	MCF-7	W138
**47a**	0.022	0.014	0.015	0.298
5-FU	0.061	0.041	0.0415	0.051

## Conclusion

Benzimidazole is a promising category of bioactive heterocyclic compound that exhibit wide variety of biological activities because of its structural similarity with the naturally occurring nucleotides and also a focusable moiety in discovery of novel drug design in medicinal field. The present review summarizes the chemistry of various substituted benzimidazole derivatives with their antiproliferative significance towards the various cancer cell lines such as HCT116, MCF7, HepG2, HeLa, A549 and A431. Benzimidazole has established huge alertness in current time and is extremely significant heterocyclic pharmacophore in recent drug innovation and medicinal chemistry.

## Data Availability

Not applicable.

## Abbreviations

SRB: sulforhodamide B
MTT: 3-(4,5-dimethylthiazol-2-yl)-2,5-diphenyl tetrazolium bromide
EAC: Ehrlich Ascites Carcinoma
LDH: lactate dehydrogenase
5-FU: 5-fluorouracil
µM: micro mole
NSCLC: non-small-cell lung carcinoma
CC: colon cancer
CNSC: central nervous system cancer
OC: ovarian cancer
PC: prostate cancer
BC: breast cancer
RC: renal cancer
MCF7: breast adenocarcinoma 7
HCT116: human colorectal carcinoma
DMSO: dimethyl sulfoxide
